# Translational Application of Recombinant Humanized Type III Collagen in Facial Rejuvenation: A Randomized Controlled Trial

**DOI:** 10.1111/jocd.70529

**Published:** 2025-11-09

**Authors:** Zeyu Huang, Ruzhi Zhang, Minmin Sheng, Ting Tian, Bin Xu, Fang Wang, Danmin Liu

**Affiliations:** ^1^ Department of Dermatology Changzhou First People's Hospital Changzhou China; ^2^ Department of Dermatology The Second Affiliated Hospital of Wannan Medical College Wuhu China

**Keywords:** clinical trial, injectable aesthetic, recombinant humanized type III collagen, skin aging

## Abstract

**Background:**

Skin aging is a common aesthetic concern that drives the demand for new therapeutic strategies. Lyophilized injectable recombinant humanized type III collagen (rhCol III) is an emerging biomaterial with promising regenerative potential; however, clinical data on its efficacy is limited. This study aimed to evaluate the safety and effectiveness of rhCol III in improving the signs of skin aging.

**Methods:**

A total of 55 participants were enrolled in the study. Those in the treatment group received “water‐light” intradermal injections of the study product alongside their routine skincare regimen. The control group continued with the routine skincare regimen alone. Clinical efficacy was assessed after treatment using VISIA imaging, evaluations by professional dermatologists, and self‐assessments by the participants.

**Results:**

Of the 52 participants who completed the follow‐up (39 in the treatment group, 13 in the control group), scores on the Global Aesthetic Improvement Scale (GAIS) improved significantly in the treatment group at day 90 ± 7 (from 3.17 ± 0.39 to 2.97 ± 0.58), with no notable change in the control group. CMH chi‐square analysis revealed a significant difference in response rates (71.8%; 95% CI: 54.2%–89.4%). VISIA analysis showed significant improvements in spots, wrinkles, texture, and porphyrins (corrected *p* < 0.00625). Generalized estimating equation (GEE) modeling supported a time‐dependent cumulative effect. The treatment was well tolerated, with stable visual analogue scale (VAS) scores and no serious adverse events.

**Conclusion:**

This preliminary study indicates that lyophilized rhCol III can significantly improve the appearance of the skin and is safe to use.

## 1 Introduction

Skin aging compromises the protective function of the skin barrier, posing a significant aesthetic concern for those seeking beauty, and rejuvenation. The development of skin aging is influenced by both intrinsic factors such as genetic predisposition and metabolic alterations, as well as extrinsic environmental factors including ultraviolet radiation, smoking, air pollution, and poor nutrition [1]. Collectively, these factors induce cellular senescence and are closely associated with elevated oxidative stress levels in the body [2, 3, 4, 5]. The accumulation of reactive oxygen species (ROS) induced by UV radiation is widely recognized as a central mechanism driving photoaging. ROS can disrupt epidermal and dermal structures, impair barrier function, and activate multiple cellular signaling pathways, thereby altering gene expression profiles involved in skin aging [2, 6]. A growing body of clinical research is now directed towards antioxidant interventions to delay skin aging.

Key challenges in aesthetic and plastic medicine include effectively addressing the signs of facial aging, alleviating skin dryness, and slowing down the aging process. In recent years, aesthetic medicine has gained global popularity. Current anti‐aging strategies include topical pharmacological treatments [7, 8, 9], laser therapy, injectable biostimulatory agents, and cosmetic surgical procedures. Of these, injectable therapies are favored due to their minimally invasive nature, ease of administration, and short recovery times. However, the efficacy and safety of commonly used injectable products are still being debated, particularly with regard to potential long‐term risks. Therefore, there is an urgent need for new injectable materials that are both safe and effective.

Collagen is a key structural protein responsible for the strength and elasticity of the skin. It has therefore attracted significant interest in skin rejuvenation research. A reduction in types I and III collagen, coupled with the functional degradation of elastic fibers in the dermis, contributes to skin laxity and visible aging [10]. Several studies have described the molecular mechanisms and pathways that promote neocollagenesis and elastin production within the dermal layer [11, 12]. Many of these technologies are based on the principle of dermal remodeling following mechanical or thermal injury [13]. While the potential benefits of oral collagen supplementation on skin health have been investigated [14], clinical research on direct collagen injection remains limited.

Recombinant humanized type III collagen (rhCol III) lyophilized fibers are a novel aesthetic biomaterial with excellent moisturizing and hydrating properties, offering a promising approach to improving skin condition. However, their clinical efficacy and safety have yet to be systematically validated. This study aimed to conduct a prospective clinical trial to comprehensively evaluate the effectiveness and safety of intradermal injection of rhColIII in improving skin condition.

## 2 Methods

### 2.1 Study Design

This study was designed as a prospective, randomized, blank‐controlled, superiority clinical trial. Ethical approval for this study was obtained (Approval No. 2022CL018‐01). The study was conducted in accordance with the principles of the Declaration of Helsinki, and approval was obtained from the institutional ethics committee prior to participant enrollment. Inclusion criteria included healthy individuals aged 18–65 years with Fitzpatrick skin types II–V who exhibited at least two clinical signs of facial skin aging, such as dryness, roughness, fine lines, enlarged pores, or a dull complexion. Exclusion criteria included: (1) known allergies to local anesthetics or collagen; (2) receiving any treatments within the past 6 months that might interfere with efficacy evaluation (e.g., light‐ or laser‐based therapies); (3) severe facial infections, scars, tattoos, or significant systemic diseases; and (4) pregnancy or planning to become pregnant during the study period.

**FIGURE 1:**
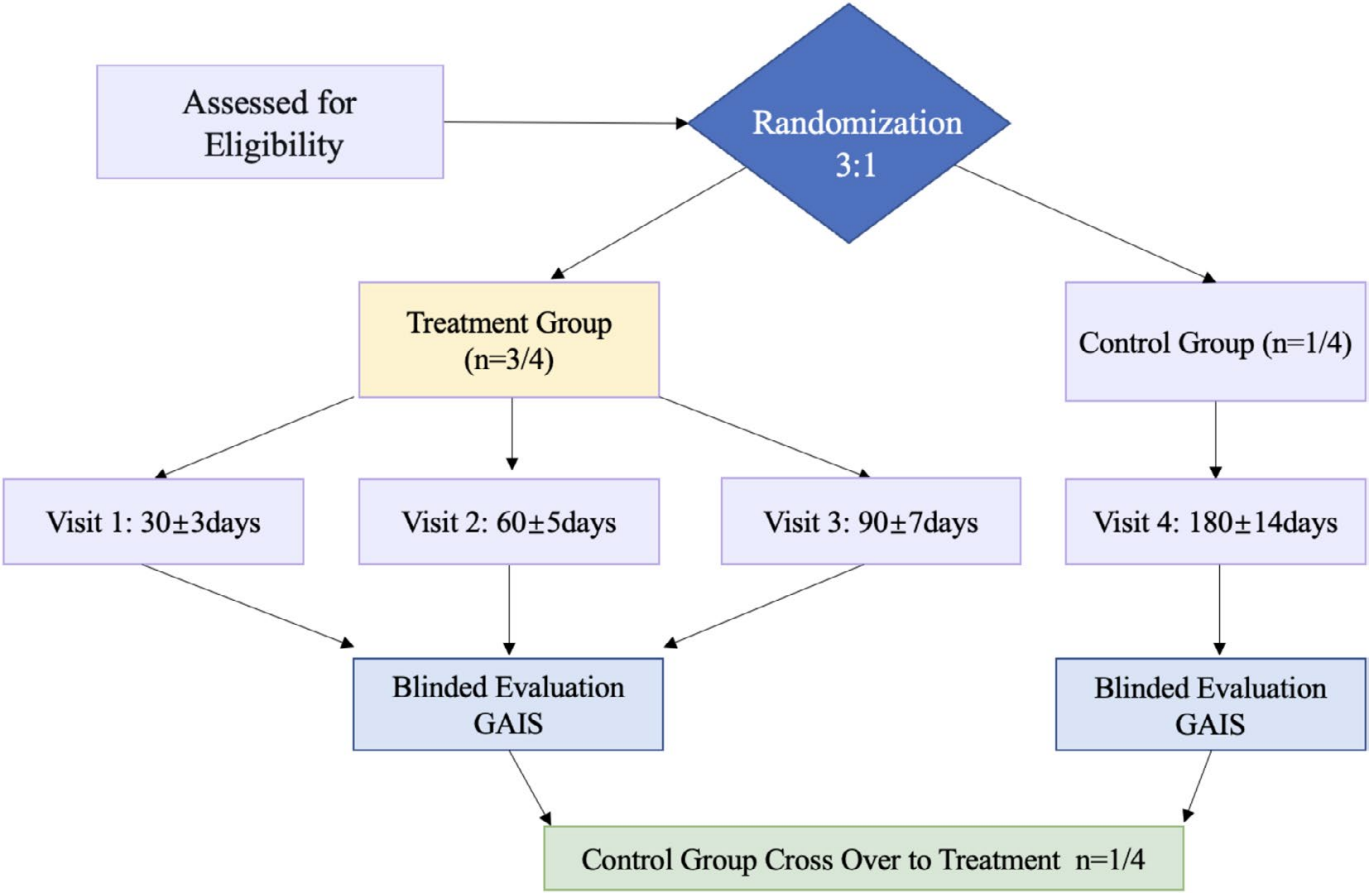
Flowchart of the clinical trial design. This summarizes the screening, enrolment, and effectiveness assessment processes for a study investigating the efficacy of rhCol III in facial rejuvenation.

Eligible participants who provided written informed consent were randomly assigned to the treatment or control group at a ratio of 3:1. Figure 1 summarized the overall flow of participants through the study.

### 2.2 Sample Size Estimation

Based on preliminary trial data, the expected GAIS improvement rate was set at 70% for the experimental group and 30% for the control group with a superiority margin of 20%. Using two‐sided *α* parameters of 0.05 and 80% statistical power parameters, the sample size calculation indicated that 64 participants were required (32 per group). Taking into account ethical constraints (to minimize the duration of non‐interventional observation for participants) and the practical conditions of a single‐center study, 39 participants were ultimately enrolled in the experimental group and 13 in the control group. A post hoc power analysis showed that, based on the observed effect size (Cohen's *h* = 1.32), the current sample size achieved > 99.9% statistical power. This indicates the sample size is adequate to detect significant between‐group differences.

### 2.3 Intervention and Follow‐Up

Prior to treatment, participants in the treatment group were given topical anesthetic in the form of lidocaine cream, which they applied for 1 h. A syringe preloaded with 3 mL of rhCol III lyophilized fibers was connected to the standard treatment head. An experienced physician adjusted the treatment parameters based on the participants' facial characteristics and administered injections in a seamless, dense, “carpet‐like” pattern to ensure uniform coverage. Post‐treatment care included advice on sun protection. The intervention was administered three times at four‐weekly intervals. The control group received “routine skincare only” (including a gentle cleanser and moisturizer) rather than a placebo. This approach was adopted due to the pragmatic design of the study, which aimed to evaluate the incremental benefit of the target treatment compared to standard care. This approach also avoided the methodological challenges of developing a credible placebo for the device, while ensuring that all participants received basic care that was ethically appropriate.

Follow‐up visits were scheduled 30, 60, 90, and 180 days after the initial treatment to evaluate improvement in skin condition, as well as any adverse events (AEs) or serious adverse events (SAEs). Concomitant medication use and device‐related issues were also evaluated. Participants in the control group did not receive any treatment on the day of randomization, but underwent the same evaluations as the treatment group at days 30, 60, and 90. Following the day 90 evaluation, the control group received the same treatment regimen as the treatment group, followed by the relevant follow‐up assessments.

### 2.4 Outcome Assessment and Data Anonymization

Participants were randomized (3:1) to the test or control group using a computer‐generated sequence, which was concealed in sequentially numbered, opaque, sealed envelopes (SNOSE). Although the injectors and participants were unblinded, a rigorous blinded endpoint assessment was enforced. Standardized clinical photographs, labeled only with a unique study code to anonymize data, were evaluated by two independent assessors who were not involved in patient treatment and were blinded to group allocation and visit sequence. Standardized facial images were used during the assessments, with each expert scoring the overall aesthetic improvement using the Global Aesthetic Improvement Scale (GAIS). The final score was calculated as the mean of the three ratings. The scoring criteria were defined as follows: 1 = very much improved; 2 = much improved; 3 = moderately improved; 4 = no noticeable change; 5 = worsened compared to baseline [15].

### 2.5 Outcome Measures

1. Treatment efficacy (GAIS score): Following the first treatment/randomization, both the patient and the investigator provided evaluations using the GAIS at the 90 ± 7‐day assessment. The mean score was calculated, and treatment efficacy was defined as the proportion of subjects with a GAIS score between 1 and 3. The efficacy rate was calculated as follows: Efficacy rate = Number of subjects with GAIS scores of 1, 2, or 3/Total number of subjects evaluated × 100% [15]
2. GAIS score at 180 ± 14 days: Blinded evaluators provided GAIS scores and grade distributions at 180 ± 14 days after the initial treatment/randomization.
3. VISIA professional facial image analysis: Changes in skin parameters, including spots, wrinkles, texture, pores, UV spots, brown spots, red areas, and porphyrins, were analyzed using VISIA imaging by comparing post‐treatment values with baseline values.
4. Assessment of skin roughness: Blinded evaluators rated skin roughness using a standardized scale (Table 1) at baseline (day 0), 90 ± 7 days and at the corresponding post‐treatment time points.
5. Evaluation of pore size: Pore enlargement was assessed using a standardized reference chart (Table 2) at the same time points as above.
6. Assessment of skin tone and glow satisfaction: Overall satisfaction with skin tone and radiance was evaluated using a standardized satisfaction score, and the total satisfaction rate was calculated for each group. Subject satisfaction was assessed using a five‐point scale ranging from 1 (very dissatisfied) to 5 (very satisfied). The proportion of subjects who rated their satisfaction as 4 or 5 was defined as the overall satisfaction rate.
7. Safety endpoints: Any adverse reactions following each treatment, including bleeding, edema, and scaling, were systematically recorded. Additionally, pain intensity was evaluated using a VAS score within 30 min after treatment. A score of 0 indicated no pain; a score of 1–3 indicated mild pain, a score of 4–6 indicated moderate pain, and a score of 7–10 indicated severe pain.

**TABLE 1:** Skin surface roughness scale [16].

Grade	Terminology	Description
0	Absent	Skin is smooth
1	Minimal	Slight roughness and uneven skin texture
2	Mild	Moderate roughness and uneven skin texture, with possible early actinic elastosis
3	Severe	Severe roughness and uneven skin texture, crosshatched fine lines, noticeable actinic elastosis
4	Extreme	Severely rough skin and texture with prominent actinic elastosis

**TABLE 2:** Facial pore enlargement severity reference scale [17].

Grade	Severity level	Description
1	None	Pores are rarely visible
2	Mild	Slightly visible pores
3	Moderate	Clearly visible pores
4	Moderate–severe	Prominently visible pores or pores containing comedones smaller than pore size
5	Severe	Very prominent pores or pores containing comedones matching the pore size
6	Extreme	Significantly enlarged pores or pores with protruding comedones giving a strawberry‐like appearance

### 2.6 Statistical Analysis

All statistical analyses were performed using SPSS version 23.0. Continuous variables were first tested for normality. Those that followed a normal distribution were expressed as mean ± standard deviation (*x* ± *s*) and analyzed using one‐way repeated measures ANOVA. Data that did not follow a normal distribution were presented as quartiles and analyzed using paired rank sum tests. A *p*‐value of < 0.05 was considered statistically significant.

## 3 Results

### 3.1 Demographic Data and Baseline Characteristics

A total of 55 subjects were screened for this clinical trial. Of these, 52 completed the primary endpoint evaluation (one subject discontinued the trial after reaching this point). Of these, 39 were assigned to the treatment group and 13 to the control group. Table 3 shows the baseline distribution of the selected subjects' age, height and weight, and there is no significant difference among these three variables (*p* > 0.05), making them suitable for subsequent effect analysis.

**TABLE 3:** Baseline demographic characteristics of the study participants.

Characteristic	Treatment group (*n* = XX)	Control group (*n* = XX)	*p*
Age (years)			0.38
Mean ± SD	38.18 ± 9.27	36.08 ± 9.27	
Median (min–max)	36.00 (24.00–59.00)	35.00 (28.00–45.00)	
Height (cm)			0.59
Mean ± SD	161.46 ± 5.43	160.62 ± 4.68	
Weight (kg)			0.82
Mean ± SD	56.67 ± 6.85	56.35 ± 7.26	
Median (min–max)	57 (48–75)	55 (46–70)	

*Note:* The data are presented as mean ± standard deviation or median (minimum–maximum). *p*‐values were calculated using an independent samples *t*‐test for age and height, and Mann–Whitney *U*‐test for weight.

Despite the relatively small size of the control group, a post hoc power analysis revealed exceptionally high statistical power (> 99.9%) for the primary endpoint. Sensitivity analysis confirmed the robustness of the findings, showing that power remained above 95%, even under the conservative assumption that 20% of the control group would respond (Cohen's *h* = 1.32) (Table 4).

**TABLE 4:** Sensitivity analysis of study power under varying control group response scenarios.

Analysis scenario	Assumed control group response rate (%)	Effect size (Cohen's *h*)	Statistical power (%)	Primary endpoint *p*
Observed data	0	1.32	> 99.9	< 0.001
Conservative scenario	20	1.07	95.2	< 0.001
More conservative scenario	30	0.86	82.1	0.003

### 3.2 Efficacy Evaluation

#### 3.2.1 Time Trend Analysis of GAIS

This study used the GAIS to evaluate aesthetic improvements in subjects before treatment and during the follow‐up period. The summarized data for all groups is presented in Table 5. In the treatment group, the GAIS score decreased gradually from 3.17 ± 0.39 at baseline to 2.97 ± 0.58 at the final follow‐up, indicating a significant improvement in aesthetic outcomes following treatment. By contrast, GAIS scores in the control group increased slightly (from 3.88 ± 0.30 to 3.96 ± 0.14) in the absence of any intervention, suggesting that aesthetic outcomes may remain unchanged or even deteriorate without treatment.

**TABLE 5:** GAIS scores (mean ± SD).

Group	GAIS1 (30 days)	GAIS2 (60 days)	GAIS3 (90 days)	Statistics
Treatment group (*n* = 39)	3.17 ± 0.39	3.08 ± 0.48	2.97 ± 0.58	Fisher's exact test, *p* < 0.001
Control group (*n* = 13)	3.88 ± 0.30	3.92 ± 0.28	3.96 ± 0.14	

#### 3.2.2 Treatment Efficacy Rates: A Comparison Between Groups

After adjusting for center effects, the CMH chi‐square test revealed a statistically significant difference in rates of improvement in skin condition between the treatment and control groups at 90 ± 7 days post‐randomization (Table 6). The difference in response rates was 71.8% (95% CI: 54.2%–89.4%). As the lower bound of the confidence interval (54.2%) is well above the predefined superiority margin of 20%, the primary endpoint was met. Furthermore, the treatment group showed significantly greater improvement than the control group for the secondary endpoints at days 30 (53.8% vs. 7.7%, *p* = 0.005) and 60 (56.4% vs. 0.0%, *p* < 0.001). These results suggest that the treatment group experienced a statistically significant and clinically meaningful improvement in skin condition compared to the non‐intervention group.

**TABLE 6:** A comparison of efficacy rates between different groups.

Group	Time point (days)	Effective cases/total sample	Efficacy rate (%)	Inter‐group difference (95% CI)	Test method	*p*
Treatment	30	21/39	53.8	46.1% (18.9–73.3)	Fisher's exact test	0.005
Control	30	0/13	0.0			
Treatment	60	22/39	56.4	56.4% (35.1–77.7)	Fisher's exact test	< 0.001
Control	60	0/13	0.0			
Treatment	90	28/39	71.8	71.8% (54.2–89.4)	CMH chi‐square test	< 0.001
Control	90	0/13	0.0			

#### 3.2.3 Longitudinal Efficacy Trend Analysis

GEE analysis revealed a significant improvement in GAIS scores over time within the treatment group (group × time interaction: *β* = 0.89, *p* < 0.001) (Table 7). This suggests a cumulative therapeutic effect throughout the course of treatment.

**TABLE 7:** Longitudinal efficacy trend analysis.

Variable	Coefficient (*β*)	Standard error (SE)	*z*‐value	*p*	95% CI
Group (treatment)	2.15	0.48	4.48	< 0.001	1.21–3.09
Time (per 30 days)	0.62	0.17	3.65	< 0.001	0.29–0.95
Group × Time interaction	0.89	0.22	4.05	< 0.001	0.46–1.32

### 3.3 VISIA Scores

As shown in Table 8, composite VISIA scores at baseline and after treatment showed that the treatment group experienced significantly greater improvements in terms of spots, wrinkles, texture, and porphyrins than the control group (*p* < 0.00625). However, improvements in pores and UV spots did not reach statistical significance after correction for multiple comparisons. This suggests that further research is needed to validate these findings in a larger sample. Representative follow‐up images from selected participants are shown below.

**TABLE 8:** A comparison of VISIA scores before and after treatment.

Parameter	Treatment group median difference (IQR)	Control group median difference (IQR)	Inter‐group *p*‐value (Mann–Whitney *U*)	Adjusted *p*‐value	Effect size (Cliff's delta)	Significance (*α* = 0.00625)
Spots	2.1 (0.8, 4.3)	0.5 (−0.3, 1.2)	< 0.001	0.008	0.62	Significant*
Wrinkles	3.5 (1.2, 5.7)	0.8 (−0.1, 1.5)	< 0.001	0.008	0.68	*
Texture	−1.2 (−2.1, −0.5)	0.1 (−0.4, 0.3)	< 0.001	0.008	0.71	*
Pores	1.8 (0.5, 3.5)	0.2 (−0.7, 0.9)	0.002	0.016	0.45	Not
UV spots	1.5 (0.3, 2.9)	0.3 (−0.2, 1.1)	0.004	0.032	0.38	Not
Brown spots	1.2 (0.1, 2.4)	0.1 (−0.5, 0.8)	0.007	0.056	0.33	Not
Red areas	0.7 (−0.2, 1.5)	0.2 (−0.4, 0.9)	0.085	0.680	0.18	Not
Porphyrins	2.4 (1.1, 4.1)	0.6 (−0.2, 1.3)	< 0.001	0.008	0.59	*

*Note:* The “*” indicates that the index is significant.

### 3.4 VAS Trend Over Time

As shown in Table 9, repeated measures ANOVA revealed a downward trend in VAS scores over the course of several treatment sessions. Notably, only one case of abnormal pain was reported, with a VAS score of 7 during the initial treatment. However, no significant pain was experienced in subsequent sessions.

**TABLE 9:** Changes in VAS scores.

VAS assessment	Mean ± SD	Median	Reduction rate
VAS1	1.51 ± 1.37	2	—
VAS2	1.21 ± 1.13	1	22.2%
VAS3	0.97 ± 0.74	1	28.9%

### 3.5 Safety Evaluation

Two mild adverse events (facial pruritus and allergic dermatitis) were reported, but the investigators deemed them unlikely to be the study treatment. Both events resolved completely. There were no device‐related malfunctions.

## 4 Discussion

Skin aging is a complex, multifactorial process driven by both intrinsic genetic factors and epigenetic influences. Clinically, it manifests as wrinkles, an uneven skin texture, changes in skin tone and pigmentary imbalances [10]. Although these changes are not life‐threatening, they can have a significant emotional and psychological burden, often leading to negative moods, and maladaptive personality traits [18].

**FIGURE 2:**
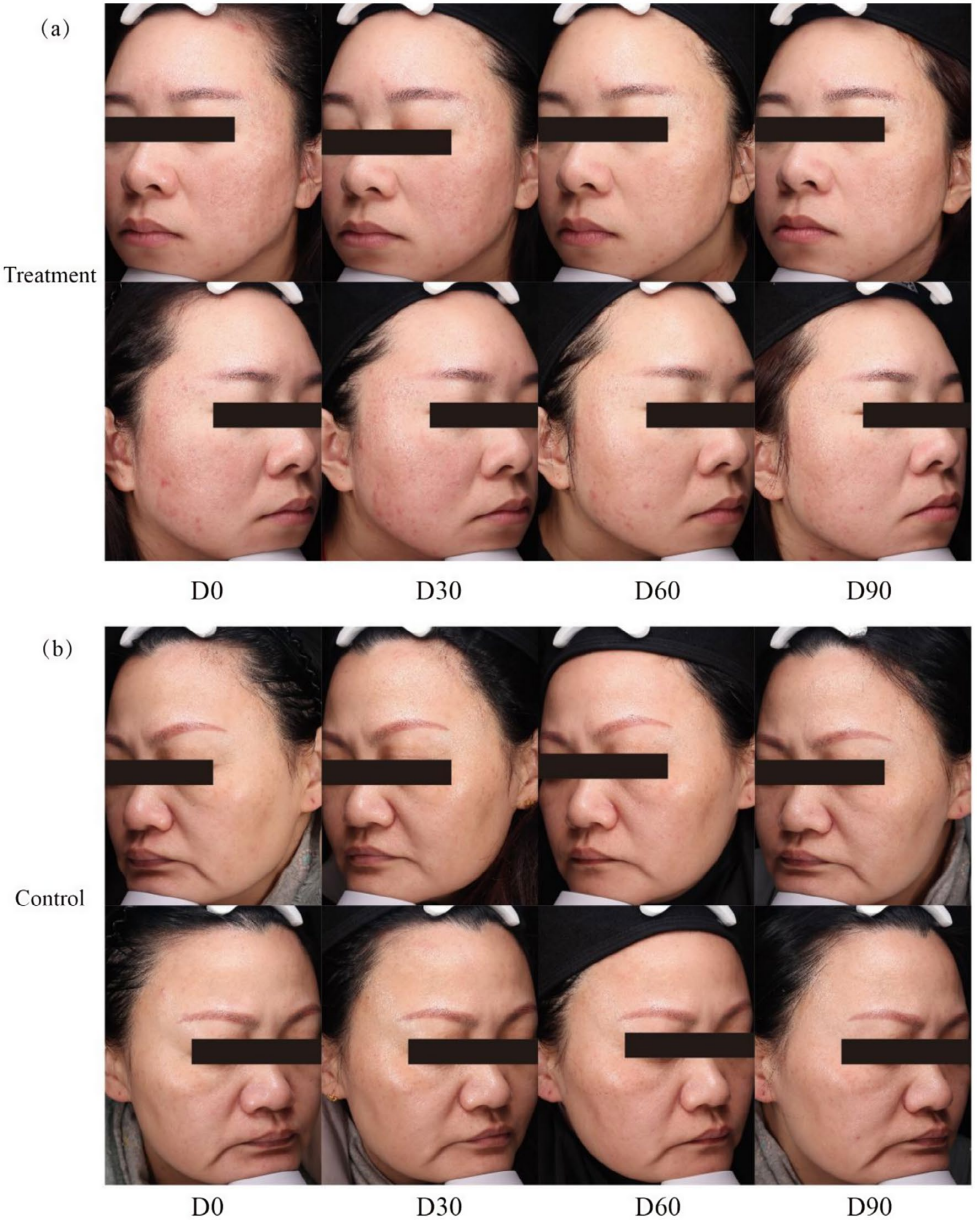
Clinical photographs of facial skin at baseline and follow‐up. (a) Patient 1: A 32‐year‐old female in the treatment group. (b) Patient 2: A 44‐year‐old female in the control group.

In this randomized controlled trial, we demonstrated that injectable rhCol III significantly improved the clinical signs of skin aging, and was well tolerated. Specifically, Figure 2 shows a clear improvement in skin texture in the before‐and‐after photographs of several patients. Ninety days after the first injection, the treatment group had achieved an efficacy rate of 71.8% in terms of skin improvement, whereas no improvement was observed in the control group. GAIS scores decreased significantly in the treatment group compared with baseline, whereas no improvement occurred in the control group, highlighting a clear benefit of the treatment. These findings are consistent with those of Tao et al. [19], who reported comparable enhancements in facial skin elasticity and smoothness subsequent to rhCol III injection. Furthermore, the time‐dependent trend observed in the GEE model suggests that rhCol III may have a cumulative effect with repeated treatments, possibly through gradual extracellular matrix (ECM) remodeling.

From a structural perspective, VISIA image analysis revealed significant improvements in spots, wrinkles, texture, and porphyrins in the treatment group. Each parameter reached or exceeded the statistical threshold after multiple comparison corrections. However, no significant changes were observed in terms of pores, UV spots or brown spots. This may be due to baseline heterogeneity, a smaller sample size or insufficient observation duration. A supplementary delayed‐intervention group showed an improvement that was intermediate between that of the early‐treatment and the untreated groups, which further confirms the positive impact of rhCol III on overall skin quality. These results align with preclinical findings indicating that rhCol III promotes dermal fibroblast proliferation, collagen I and elastin deposition, as well as improved ECM organization in photoaged skin models [20, 21].

The mechanism underlying rhCol III‐induced rejuvenation is likely related to its ability to act as a biomimetic scaffold, supporting fibroblast adhesion and migration, while simultaneously stimulating neocollagenesis and dermal remodeling [22]. Compared to traditional fillers such as hyaluronic acid or poly‐l‐lactic acid (PLLA), rhCol III may offer superior biocompatibility and a lower risk of an inflammatory reaction, thanks to its amino acid sequence and triple‐helical structure, which closely resemble those of endogenous type III collagen [23]. These advantages could explain the sustained, natural‐looking clinical outcomes observed in this study, as well as the low incidence of adverse effects.

In terms of safety, repeated‐measures ANOVA showed that VAS pain scores remained within the range of 1–3 throughout the treatment course, demonstrating a significant downward trend. This indicates excellent tolerability and the absence of cumulative pain. Only two mild, transient adverse events were recorded, and no device defects or treatment‐related complications were observed. These results are consistent with those of recent multicenter trials demonstrating the safety and long‐term stability of injectable collagen fillers [24, 25].

While this study demonstrates the promising clinical potential of rhCol III, several limitations should be acknowledged. Firstly, the sample size was relatively small and the follow‐up period was limited. Secondly, imaging or histological assessments were not incorporated into the study to directly verify collagen neogenesis. Future investigations should be conducted as larger multicenter trials that incorporate high‐resolution skin ultrasound or biopsy analyses to objectively evaluate dermal regeneration. Such studies should also include additional quantitative and objective measurements, such as skin elasticity, hydration, and sebum levels, in order to develop a more comprehensive framework for assessing efficacy.

In conclusion, this study provides clinical evidence that supports the efficacy and safety of using rhCol III for facial rejuvenation. The significant improvement in skin texture, wrinkles and radiance, coupled with excellent patient tolerance, establishes rhCol III as a promising next‐generation biotherapeutic for aesthetic dermatology and regenerative skin applications.

## Author Contributions

Zeyu Huang: conceptualization, methodology, investigation, formal analysis, writing – original draft, writing – review and editing. Minmin Sheng: experimental investigation, data curation, formal analysis, writing – review and editing. Ting Tian: experimental investigation, data curation, writing – review and editing. Bin Xu: experimental investigation, data curation, writing – review and editing. Fang Wang: experimental investigation, data curation, writing – review and editing. Danmin Liu: experimental investigation, writing – review and editing. Ruzhi Zhang: conceptualization, funding acquisition, resources, supervision, project administration, validation, writing – review and editing (final approval and corresponding submission).

## Consent

Written informed consent was obtained from the patient for the publication of case details, imaging or videos.

## Conflicts of Interest

The authors declare no conflicts of interest.

## Data Availability

The data that support the findings of this study are available from the corresponding author upon reasonable request.
